# Social media use for work during non-work hours and turnover intention: the mediating role of burnout and the moderating role of resilience

**DOI:** 10.3389/fpsyg.2024.1391554

**Published:** 2024-07-31

**Authors:** Zhenbang Fang, Yuanjie Bao, Min Hua

**Affiliations:** School of Public Administration and Policy, Renmin University of China, Beijing, China

**Keywords:** social media use, resilience, turnover intention, burnout, job demands–resources (JD-R)

## Abstract

**Introduction:**

This study uses survey data from 504 Chinese teachers to investigate the relationship between social media use for work during non-work hours (SMUNW) and turnover intention, focusing on the mediating role of burnout and the moderating role of resilience in this relationship.

**Methods:**

In November 2023, online survey links were sent to 529 teachers from Shandong Province, China, asking them to report their perceptions of SMUNW, burnout, resilience, and turnover intention. A sample of 504 valid responses was obtained. The analysis was performed using SPSS 26.0 and Hayes’ PROCESS MACRO for SPSS for testing the hypotheses.

**Results:**

The results revealed that burnout acts as a mediator in the relationship between SMUNW and turnover intention. In addition, resilience moderated the relationship between SMUNW and burnout such that when resilience was higher, the relationship between SMUNW and burnout was alleviated. Moreover, resilience moderated the indirect relationship between SMUNW and turnover intention through burnout such that when resilience was higher, the indirect relationship was alleviated.

**Discussion:**

The results of this study indicate that SMUNW is related to turnover intention through the mediating role of burnout. Furthermore, resilience moderates the influence of SMUNW on burnout and thus weakens the influence of SMUNW on turnover intention through burnout. This study expands our knowledge of the nuanced influence mechanisms of social media use in the context of increasing technostress among public employees. Practically, it suggests that managers should pay due attention to the impairments brought about by social media use, especially from the perspectives of preventing burnout and fostering resilience.

## 1 Introduction

With the rapid development of information and communication technology (ICT), the use of social media has become increasingly common (Leftheriotis and Giannakos, 2014; Andreassen et al., 2017). Employees are not only required to use social media during working hours (Yu et al., 2018; Cao and Yu, 2019; Rozgonjuk et al., 2020) but are also required to continue using social media to handle work matters during non-work hours (Masood et al., 2023; Wang and Li, 2023).

Using social media for work during non-work hours (SMUNW) is prevalent (Yang et al., 2023) because it can bring benefits to both the organization and its employees. For example, SMUNW can enhance communication and collaboration and thus improve efficiency (Jong et al., 2021). It also boosts work engagement by keeping employees closely connected (Bodhi et al., 2023). Additionally, SMUNW offers flexibility, allowing employees to manage tasks and address urgent issues outside traditional work hours, thereby maintaining productivity during non-work hours (Oksa et al., 2022). However, SMUNW might also lead to negative consequences. Technostress is a kind of stress experienced by individuals due to overuse of ICT and may lead to impairments (Tarafdar et al., 2019). Among its various forms, technostress is particularly salient when the use of technology extends work demands into personal times (Brooks and Califf, 2017). SMUNW, as a form of extended work demand through social media, can significantly contribute to technostress in this regard (Maier et al., 2015). For example, continuing to use social media to handle work during non-work hours takes up time that can be devoted to family issues, resulting in more work–family conflicts (Yue, 2022, 2023; Wang and Li, 2023). Additionally, an online survey of employees in the U.S. conducted by Yue (2022) showed that SMUNW reduces employee engagement at work. Wang and Li (2023) conducted a study among Chinese civil servants and reached similar conclusions. Moreover, it was also reported that SMUNW could lead to turnover intention (Tang et al., 2020; Sun et al., 2024).

Given the ubiquity of SMUNW in today’s working environment and many of its negative consequences, it is critical to find countermeasures to mitigate its impairments. Unfortunately, very limited empirical effort is devoted to this purpose (Wang and Li, 2023). Recognizing this gap, this study explores the role of resilience in buffering the adverse consequences of SMUNW. Resilience was chosen because of the following reasons. First, resilience has been shown to help individuals cope with stress and recover from adverse situations, making it a suitable buffer against the negative impacts of continuous work demands (Ross et al., 2023). Second, resilience enhances an individual’s ability to maintain psychological wellbeing and productivity under stress, which is crucial when dealing with the constant connectivity required by SMUNW (Wang et al., 2023). Third, incorporating resilience allows us to explore how personal characteristics can influence the effects of SMUNW, providing a more comprehensive understanding of its impacts and potential interventions (Wu et al., 2020). Specifically, the research question of this study is as follows: Is SMUNW related to turnover intention? If so, does burnout mediate this relationship? Can resilience moderate the impact of SMUNW on burnout and thus mitigate its influence? Empirically, this study surveys 504 teachers from Shandong Province of China to provide answers to these questions. Hierarchical regression and bootstrapping methods were used to test the hypothesized moderated mediation model.

This study aimed to contribute in three ways. First, we investigate whether SMUNW is related to the turnover intention among Chinese teachers. This profession relies heavily on social media during both work and non-work hours. In China, it is common for teachers to use applications such as WeChat and DingTalk to communicate with students and parents and handle various teaching and management tasks after normal working hours (Wan et al., 2016). These applications and platforms provide instant messaging, video conferencing, and file-sharing functions, making it convenient for teachers to handle work matters (Liu et al., 2021). However, this usage also blurs the boundaries between work and life, increasing the risk of experiencing technostress and related burnout (Basuni and Sopiah, 2023). Previous work on SMUNW mostly focused on civil servants (Wang and Li, 2023) or employees from various private organizations (Yue, 2022). SMUNW study among teachers is scant. To this end, the current study is expanding the empirical base of SMUNW scholarship. Second, we delineate the effect mechanism of SMUNW by incorporating the important mediating role of burnout. By elucidating the effect mechanism of SMUNW through burnout, we can understand how SMUNW influences turnover intention by influencing a more proximal psychological state, namely, burnout. This will provide important theoretical and practical implications within a context, whereby social media has blurred the boundary between work and family. Third, by exploring the moderating role of resilience, we can clarify the boundary conditions of SMUNW from the perspective of individual positive psychological resources, which is an important supplement to the current limited research on SMUNW’s boundary conditions.

## 2 Theoretical framework and hypotheses

### 2.1 Theoretical background: job demands–resources theory

In recent developments, scholars interested in social media use are increasingly applying the job demands–resources (JD-R) theory to investigate its influences (Wang et al., 2021; Kalra et al., 2023; Reimann et al., 2023). This is because it is a unified theory that integrates various job demands and resources to help understand the motivational mechanism influencing employees’ attitudes and behaviors. Originating from research on burnout, the JD-R theory categorizes all aspects of the working environment into two aspects, namely, job demands and resources (Demerouti et al., 2018). Job demands are physical, psychological, social, or organizational aspects of a job that require sustained physical, cognitive, and emotional efforts (Demerouti et al., 2001), while job resources are physical, psychological, social, or organizational motivating potentials of a job that can help individuals to achieve work goals and stimulate individual learning and personal growth (Bakker and Demerouti, 2017). Although job demands often lead to strain and impairments, job resources can lead to learning and personal growth (Xanthopoulou et al., 2010). This is because job demands will consume individual resources, induce burnout, and create health problems; job resources, on the other hand, can meet individual needs and increase work motivation, which is helpful in improving individual work performance (Bakker and Demerouti, 2017). Additionally, the JD-R theory proposes that there is an interaction effect between job demand and job resources; that is, job resources can buffer the possible detrimental influences of job demands. When employees possess high levels of resources, they can better deal with job demands and maintain a higher level of motivation and wellbeing; that is, job resources protect employees from job demands and promote work motivation that leads to positive attitudes and behaviors (Demerouti et al., 2011).

SMUNW can be treated as a type of job demand that might influence employees’ turnover intention. This is consistent with previous investigations that found that SMUNW can lead to detrimental outcomes (Tang et al., 2020; Yue, 2022, 2023; Wang and Li, 2023; Sun et al., 2024), including turnover intention (Tang et al., 2020; Sun et al., 2024). Furthermore, the JD-R theory originated from the study of burnout and proposed it as the mechanism mediating job demands and resources’ influences over attitudes and behaviors. As such, this study uses burnout as a mediating variable to explore the process by which SMUNW affects teachers’ turnover intention. Additionally, resilience is treated as a job resource that may attenuate the detrimental influence of SMUNW. In summary, we ground our hypothesis development based on the JD-R theory, consistent with previous studies of SMUNW. In this regard, we tailor the JD-R theory to our research context, highlighting how burnout and resilience help explain the influence mechanism between SMUNW and turnover intention in the Chinese education sector.

### 2.2 Hypotheses development

#### 2.2.1 SMUNW and turnover intention

SMUNW was defined as “social media use for work during non-work hours” (Wang and Li, 2023, p. 2). Due to the popularity of instant messaging, employees not only use social media extensively during working hours but are also often required to use social media to stay in touch and handle work matters during non-work hours. It was also reported that the use of social media at work can cause stress to employees and lead to turnover intention (Bizzi, 2018; Cho et al., 2019; Califf et al., 2020; Harris et al., 2022; Sparidans et al., 2023).

We can speculate that continuing to use social media to handle work matters after work is even more detrimental for teachers. First, according to the JD-R theory, SMUNW can be categorized as a work demand that will bring negative emotional and cognitive reactions to teachers. To deal with these demands created by SMUNW, teachers would have to invest a significant amount of their resources. As such, in order to protect valuable resources, teachers might want to distance themselves from the situation and thus develop turnover intention, that is, turnover is regarded as a self-protection mechanism in the demanding situation of SMUNW. Second, as primary and secondary school teachers teach minors, they need more care and attention. Therefore, both parents and schools require teachers to maintain close and immediate contact with them in order to understand each student promptly and accurately. Therefore, SMUNW can be even overwhelming for teachers and may lead to the idea of leaving the teaching profession.

Empirical evidence supports these assertions. For example, SMUNW was reported to lead to turnover intention among active WeChat users (Sun et al., 2024) and employees of a pharmaceutical company (Tang et al., 2020). Based on these theoretical arguments and empirical evidence, we hypothesize the following:

*H1:* SMUNW is positively associated with turnover intention.

#### 2.2.2 The mediating role of burnout

Burnout is a prolonged response to chronic emotional and interpersonal stressors on the job (Maslach et al., 2001). The JD-R theory suggests that job demands require employees to invest resources to successfully deal with them. These demands can lead to employee burnout by taxing employees both physically and psychologically. In this regard, SMUNW was a form of job demand that would necessitate psychological and physical investments, thereby producing burnout, that is, to handle SMUNW, employees would invest a significant amount of resources that could otherwise be used to boost motivation. High job demands, such as SMUNW, can deplete resources that are otherwise used to maintain high levels of engagement, ultimately leading to burnout (Bakker and Demerouti, 2017). Research indicates that engagement and burnout are negatively correlated, and they are influenced by similar job demands and resources (Schaufeli and Taris, 2014). Specifically, when job demands are high, resources are exhausted, reducing engagement and increasing the risk of burnout (Molino et al., 2020). Therefore, it is possible that SMUNW would consume resources and increase burnout. Empirical evidence supports this position. For example, it was reported that social media use had a positive impact on burnout (Charoensukmongkol, 2016; Han et al., 2020; Liu and Ma, 2020; Nam and Kabutey, 2021). Meanwhile, studies that follow the JD-R theory have consistently reported relationships between various types of job demands and work engagement (Mauno et al., 2007; Inoue et al., 2014; Demerouti, 2018). It is thus proposed as follows:

*H2:* SMUNW is positively associated with burnout.

The JD-R theory proposes that emotional reactions would mediate the relationship between job demands and employee attitudes (Bakker and Demerouti, 2017). In this regard, burnout could be a reason how job demands, such as SMUNW, influence employee attitudes, such as turnover intention, that is, burnout is regarded as a mediating variable that explains the mechanism of the impact of SMUNW on turnover intention (Bakker and Demerouti, 2017). When job demands are too high to handle, employees cannot devote themselves fully to work roles and thus develop negative attitudes and behaviors. Studies linking burnout and turnover intention and verifying their relationship are relatively common (Janssen et al., 1999; Han et al., 2016; Lu and Gursoy, 2016; Lee, 2019). Meta-analytic evidence suggests that there is a strong positive relationship between burnout and turnover intention (Chen et al., 2022; Özkan, 2022). Therefore, it is hypothesized as follows:

*H3:* Burnout mediates the relationship between SMUNW and turnover intention.

#### 2.2.3 The moderating role of resilience

Psychological resilience is defined as a person’s ability to recover, bounce back, adjust, and even thrive after misfortune, change, or adversity (Garcia-Dia et al., 2013). Recently, stress literature is beginning to consider resilience as an important boundary condition when examining the influence of work demands (Palm-Fischbacher and Ehlert, 2014; Cole et al., 2015; García-Izquierdo et al., 2018; Stanley et al., 2021). However, there is currently no research exploring the moderating role of resilience on the relationship between SMUNW and variables such as burnout and turnover intention.

The buffering hypothesis of the JD-R theory suggests that job resources could attenuate the influence of job demands. Hobfoll’s conservation of resources (COR) theory posits that resources are valuable entities that help individuals cope with stress and challenges (Hobfoll et al., 2018). These resources include physical, social, and psychological resources, and resilience fits this definition. Additionally, resilience is considered to be an important part of personal psychological capital (Luthans et al., 2015), forming the critical basis of personal resources. Therefore, this study regards resilience as a form of personal psychological resource. Specifically, we treat resilience as an important form of job resource that employees can utilize when experiencing SMUNW, thus attenuating its detriments. On the one hand, when employees have high resilience, they may experience smaller psychological fluctuations when facing situations in which they still need to use social media to handle work outside of working hours. On the other hand, SMUNW can be seen as a type of adversity, and in this situation, employees with a higher level of resilience can recover more easily and quickly. In addition, psychological resilience can instill the belief in employees that they can handle the work demands associated with SMUNW. As such, we propose that resilience can be seen as an important form of job resource that buffers the influence of SMUNW, thereby reducing its impact on burnout. It is hypothesized as follows:

*H4:* Resilience moderates the relationship between SMUNW and burnout, such that the relationship is weaker when resilience is high.

Based on the derivation of the above assumptions, this study further proposes a moderated mediation hypothesis, that is, the indirect relationship between SMUNW and turnover intention through burnout will be moderated by resilience. It is hypothesized as follows:

*H5:* Resilience moderates the indirect relationship between SMUNW and turnover intention through burnout, such that the indirect relationship is weaker when resilience is high.

**Figure 1:**
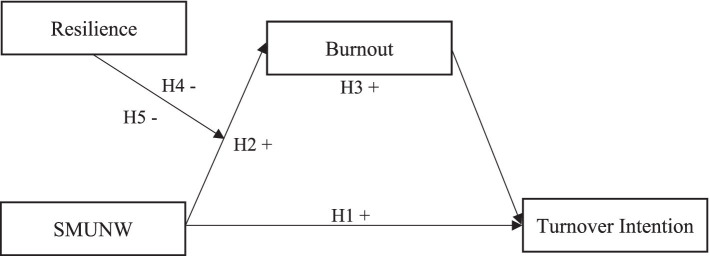
Research model.

All proposed hypotheses are depicted in Figure 1.

## 3 Methods

### 3.1 Data and samples

To test the hypotheses, we surveyed teachers from Shandong Province of China. Shandong Province is one of the provinces with the largest number of students in China. Due to the large number of students, students face great academic pressure. Meanwhile, being the hometown of Confucius, Shandong Province emphasizes education and places high expectations on education quality. In this regard, schools have put forward higher requirements for teachers, and it is not uncommon for teachers to continue using social media to handle work after work. In November 2023, we sent online survey links to 529 teachers and obtained a sample of 504 valid responses (response rate = 95.27%) assessing their SMUNW, burnout, resilience, and turnover intention. We stated at the beginning of the questionnaire that the survey is anonymous, and the data collected will only be used for academic purposes and no personal information will be disclosed. Table 1 shows summary statistics of demographic information for our sample. As can be seen, 81.35% were female (n = 410); the average age was 35.12 years (SD = 9.18); the average tenure was 12.68 years (SD = 10.05); 73.41% (370) of the respondents were married; and 41.27% (208) of the respondents serve as head teachers (in charge of a class).

**Table 1:** Demographic characteristics of participants.

	Category	Frequency	Percent
Sex	Female	410	81.35
	Male	94	18.65
Age	21–29	175	34.72
	30–39	188	37.30
	40–49	84	16.67
	50–60	57	11.31
Tenure	1–5	147	29.17
	6–10	134	26.59
	11–15	74	14.68
	16–20	41	8.13
	21–30	64	12.70
	31–45	44	8.73
Marital status	Unmarried	130	25.79
	Married	370	73.41
	Divorce	3	0.60
	Widow	1	0.20
Head teacher or not	Head teacher	208	41.27
	Ordinary teacher	296	58.73

### 3.2 Measures

#### 3.2.1 Social media use for work during non-work hours

SMUNW was measured with four items (Wang and Li, 2023). A sample item was “I often use social media to connect with colleagues for work during non-work hours.” The participants indicated their responses on a 6-point Likert-type scale, ranging from 1 = “strongly disagree” to 6 = “strongly agree.” Cronbach’s alpha was 0.918.

#### 3.2.2 Burnout

Burnout was measured using a 5-item scale (Schaufeli, 1996). A sample item is “Work makes me feel physically and mentally exhausted.” A 6-point Likert-type scale ranging from 1 = “strongly disagree” to 6 = “strongly agree” was used. Cronbach’s alpha was 0.960.

#### 3.2.3 Resilience

Resilience was measured using six items (Luthans et al., 2007). A sample item is “Because I have been through a lot of hardships before, I am now able to survive difficult times at work.” All items were assessed on a 6-point Likert-type scale ranging from 1 = “strongly disagree” to 6 = “strongly agree.” Cronbach’s alpha was 0.771.

#### 3.2.4 Turnover intention

Three items were used to measure turnover intention (Skaalvik and Skaalvik, 2011). A sample item was “I often think of leaving the teaching profession.” The responses were evaluated on a 6-point Likert-type scale ranging from 1 = “strongly disagree” to 6 = “strongly agree.” Cronbach’s alpha was 0.932.

#### 3.2.5 Control variables

The respondents’ sex (1 = male, 2 = female), age (in years), marital status, tenure (in years), and head teacher status (0 = ordinary teacher, 1 = head teacher) were included as control variables, as they might be related to burnout and turnover intention.

## 4 Results

We used SPSS 26.0 to perform statistical analysis. Hayes’ PROCESS MARCO was used for mediation, moderation, and moderated mediation analyses.

### 4.1 Common method variance test

Perceptual survey data were suitable to measure SMUNW, burnout, resilience, and turnover intention, as these are personal experiences. Unfortunately, these types of data were sensitive to common method variance. To mitigate such bias, several precautionary measures were taken. First, only measures with well-established psychometric properties were included. Second, respondents’ voluntary participation, anonymity, and the importance of personal opinions were stressed. Despite the above measures, the data in this study are still cross-sectional in nature and may suffer from potential common method bias. Therefore, this study uses the single-factor method to test common method bias (Harman, 1976). The result of an unrotated exploratory factor analysis of all variable items showed that the total variation explained was 77.81%; of which, the first component explained 38.18%. As this did not exceed 40%, it can be concluded that the problem of common method bias in this study is not serious.

### 4.2 Measurement model

Confirmatory factor analyses were used to test for convergent and discriminant validity, and the results are reported in Table 2. As can be seen, the hypothesized four-factor model fitted the data well [χ^2^ = 355.67, df = 99, χ^2^/df = 3.59, goodness-of-fit index (GFI) =0.93, comparative fit index (CFI) = 0.97, normed fit index (NFI) = 0.96, Tucker–Lewis index (TLI) = 0.95, incremental fit index (IFI) = 0.97, and root mean square error of approximation (RMSEA) = 0.07]. In addition, we also compared the proposed model to four alternative models to test for discriminant validity. The chi-square difference test and fit indexes revealed that the hypothesized four-factor model had the best fit. Moreover, the average variance extracted (AVE) and composite reliability (CR) results are shown in Table 3. The AVE values were all higher than the standard of 0.5 suggested by Fornell and Larcker (1981). Moreover, the CR values of the four constructs ranged from 0.90 to 0.96, higher than the recommended level of 0.8 (Hair et al., 2021). Furthermore, the square roots of the AVE values were greater than the inter-construct correlations (Fornell and Larcker, 1981). These results indicated that the four-factor model achieves sufficient discriminant validity.

**Table 2:** Measurement model test results.

		χ^2^	df	χ^2^/df	RMSEA	GFI	CFI	NFI	TLI	IFI
1. Hypothesized 4-factor model	SMUNW, Burnout, Resilience, TI	355.67	99	3.59	0.07	0.93	0.97	0.96	0.95	0.97
2. Alternative 3-factor model	SMUNW, Burnout + resilience, TI	622.27	102	6.10	0.10	0.91	0.93	0.92	0.90	0.93
3. Alternative 2-factor model	SMUNW, Burnout, Resilience + TI	1180.10	104	11.34	0.14	0.84	0.86	0.85	0.80	0.86
4. Alternative 2-factor model	SMUNW + Burnout + Resilience, TI	1198.58	104	11.53	0.15	0.84	0.86	0.85	0.79	0.86
5. Alternative 1-factor model	SMUNW + Burnout + Resilience + TI	1756.41	105	16.73	0.18	0.79	0.79	0.78	0.69	0.79

*N* = 504. SMUNW, Social media use for work during non-work hours; TI, Turnover intention.

**Table 3:** AVE and CR indicator results.

Factor	AVE	CR
SMUNW	0.74	0.92
Burnout	0.82	0.96
Resilience	0.64	0.90
TI	0.83	0.94

*N* = 504. SMUNW, Social media use for work during non-work hours; TI, Turnover intention.

### 4.3 Hypotheses testing

Table 4 displays the descriptive statistics and correlations among the examined variables. The correlation patterns are consistent with our hypotheses.

**Table 4:** Means, standard deviations, and correlations among variables.

	Mean	SD	1	2	3	4	5	6	7	8	9
1. Sex	1.81	0.39	-								
2. Age	35.12	9.12	−0.19^***^	-							
3. Tenure	12.68	10.05	−0.21^***^	0.94^***^	-						
4. Marital status	1.75	0.46	−0.06	0.51^***^	0.47^***^	-					
5. Head teacher	0.41	0.49	0.06	−0.27^***^	−0.26^***^	−0.28^***^	-				
6. SMUNW	4.55	0.98	0.03	0.03	0.01	−0.07	0.18^***^	**(0.86)**			
7. Burnout	3.57	1.25	0.00	−0.01	−0.03	−0.11^*^	0.17^*^	0.38^***^	**(0.91)**		
8. Resilience	4.28	0.75	−0.09^*^	0.15^**^	0.15^**^	0.15^**^	−0.06	0.11^*^	−0.27^***^	**(0.91)**	
9. TI	3.22	1.43	0.04	−0.04	−0.02	−0.09	0.09	0.19^***^	0.64^***^	−0.31^***^	**(0.80)**

*N* = 504. Square roots of the AVE are values in parentheses along the diagonal and displayed in bold. ^*^p < 0.05, ^**^p < 0.01, ^***^p < 0.001.

SMUNW, Social media use for work during non-work hours; TI, Turnover intention.

As shown in Table 5, SMUNW is positively related to turnover intention (*β* = 0.17, *p* < 0.001, Model 2), hypothesis 1 is thus supported. This indicates that employees who use social media for work during non-work hours are more likely to consider leaving their jobs. This positive relationship highlights the potential adverse effects of blurred boundaries between work and personal life induced by social media use. Further, Model 1 shows that SMUNW is positively related to burnout (*β* = 0.37, *p* < 0.001), hypothesis 2 is thus supported. In Model 3, the effect of SMUNW on turnover intention is not significant when including burnout in the model (*β* = −0.07, *p* > 0.05), while the effects of burnout (*β* = 0.66, *p* < 0.001) are significant. We used the bootstrapping method to test for the significance of the mediating effect. The indirect effect based on 5,000 bootstrapped samples is 0.35, with a 95% bias-corrected confidence interval of [0.25, 0.45], excluding 0, hypotheses 3 is thus supported.

**Table 5:** Test results for main and mediation effects.

	Burnout	Turnover intention	
	Model 1	Model 2	Model 3
Sex	−0.01(0.14)	0.03(0.16)	0.04(0.13)
Age	0.12(0.02)	−0.16(0.02)	−0.24^*^(0.02)
Tenure	−0.10(0.02)	0.17(0.02)	0.24^*^(0.01)
Marital status	−0.09(0.13)	−0.06(0.16)	−0.01(0.13)
Head teacher	−0.02(0.11)	−0.04(0.14)	−0.02(0.11)
SMUNW	0.37^***^(0.05)	0.17^***^(0.07)	−0.07(0.06)
Burnout			0.66^***^(0.04)
*R* ^2^	0.15	0.05	0.42
∆*R*^2^			0.37^***^
*F*	14.97^***^	3.93^***^	50.72^***^

*N* = 504. Standardized coefficients are reported. Values in parentheses were standard error estimates. ^*^p < 0.05, ^**^p < 0.01, ^***^p < 0.001.

SMUNW, Social media use for work during non-work hours.

**Figure 2:**
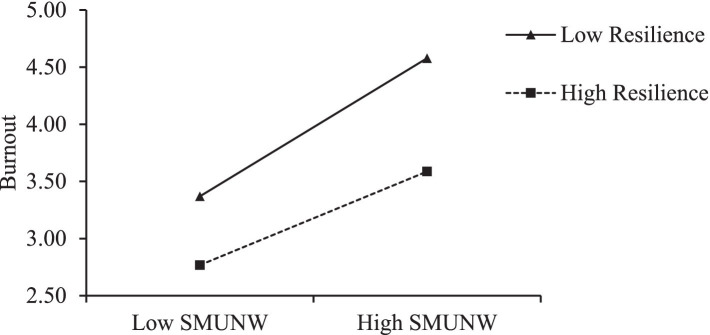
Interactive effect of SMUNW and resilience on burnout.

As Table 6 denotes, SMUNW and resilience interactively influence burnout (*β* = −0.61, *p* < 0.01, Model 5). To confirm the direction of the interaction, a simple slope was plotted at 1 standard deviation above and below the mean of resilience. As Figure 2 illustrates, the slope of the relationship between SMUNW and burnout is gentler for the high-resilience group (simple slope = 0.42) than for the low-resilience group (simple slope = 0.62). Therefore, hypothesis 4 is supported.

**Table 6:** Test results for moderation effect.

	Burnout	
	Model 4	Model 5
Sex	−0.04(0.13)	−0.04(0.13)
Age	0.13(0.02)	0.10(0.02)
Tenure	−0.09(0.01)	−0.06(0.01)
Marital status	−0.05 (0.13)	−0.05(0.13)
Head teacher	−0.02(0.11)	−0.02(0.11)
SMUNW	0.41^***^(0.051)	0.85^***^(0.21)
Resilience	−0.32^***^(0.07)	0.04(0.23)
SMUNW* Resilience		−0.61^**^(0.05)
R^2^	0.25	0.26
∆R^2^		0.01^**^
*F*	24.43^***^	21.72^***^

*N* = 504. Standardized coefficients are reported. Values in parentheses were standard error estimates. ^*^p < 0.05, ^**^p < 0.01, ^***^p < 0.001.

SMUNW, Social media use for work during non-work hours.

As Table 7 denotes, when resilience is low, the indirect effect is 0.464, with a bootstrapped 95% confidence interval of [0.354, 0555], excluding 0. When resilience is high, the indirect effect is lower at 0.314, with a bootstrapped 95% confidence interval of [0.200, 0.437], excluding 0. Furthermore, the index of the moderated mediation is 0.100, with a bootstrapped 95% confidence interval of [−0.166, −0.014], excluding 0. As such, it can be concluded that resilience moderated the indirect relationship, and Hypothesis 5 is supported.

**Table 7:** Moderated mediation results across resilience levels.

SMUNW → Burnout → TI				
	Indirect effect	BootSE	BootLLCI	BootULCI
Resilience M − 1 SD (3.531)	0.464	0.051	0.354	0.555
Resilience M + 1 SD (5.024)	0.314	0.061	0.200	0.437

Index of moderated mediation				
	Index	BootSE	BootLLCI	BootULCI
	0.100	0.038	−0.166	−0.014

*N* = 504. SMUNW, Social media use for work during non-work hours; TI, Turnover intention.

## 5 Discussion

Based on the JD-R theory, this study examines whether and how SMUNW is related to turnover intention among teachers from Shandong Province, China. We found that (1) SMUNW can increase the intention of Chinese teachers to resign; (2) burnout plays a mediating role between SMUNW and turnover intention; and (3) resilience buffers the indirect relationship between SMUNW and turnover intention through burnout. Resilience, as an important type of personal resource, buffers the impairments of SMUNW among teachers. These findings provide important theoretical and practical implications.

### 5.1 Theoretical implications

First, this study extends our knowledge related to social media use. In recent years, scholars have begun to pay attention to the use of social media and explore its impact on people. However, most research mainly focuses on non-work social media use at work (Brooks and Califf, 2017; Syrek et al., 2018), while paying little attention to SMUNW. Although these two types of social media use are both related to work attitudes and behaviors, there might be significant differences concerning the direction of their influences. For example, non-work social media use at work and SMUNW might have very different effects on employees’ work engagement. Non-work social media use at work can serve as a micro-break and increase work engagement (Syrek et al., 2018). However, using social media to deal with work after hours can have a negative impact on employee work engagement. An online survey of employees in the United States conducted by Yue (2022) showed that SMUNW reduces employee engagement when they are at work. Wang and Li (2023) conducted a study in China and reached similar conclusions. This study extends our knowledge of social media use by focusing on how SMUNW is associated with burnout and turnover intention among a sample of Chinese teachers. While public employees, such as teachers, are increasingly using social media after hours to work, the possible impairments and related mechanisms are seldom examined. As such, this study extends our understanding of the nomological network of SMUNW. In this regard, this study expands the content of social media research and provides a reference for further studies.

Second, although there have been relevant studies exploring the impact of SMUNW (Wang and Li, 2023), the discussion mainly focuses on the perspective of employee cognition and there is relatively little exploration of the mechanism of burnout. Based on the JD-R theory, SMUNW can be seen as a job demand that consumes employees’ passion, enthusiasm, and motivation, leading to burnout. Job burnout can, in turn, affect employees’ commitment and enthusiasm and increase their tendency to quit. This study reveals the fatigue mechanism of SMUNW affecting employee turnover intention, providing a new perspective for understanding the impact of SMUNW. Furthermore, by validating burnout as the mediating mechanism of SMUNW, this study successfully integrates the JD-R theory into our discussion of SMUNW. In this regard, future research can further understand the influences of SMUNW from the perspective of the JD-R theory.

Third, this study clarifies the important boundary conditions for the impact of SMUNW. According to the JD-R theory, job resources can buffer the negative impact of job demands. Resilience, as an important type of personal resource, can be regarded as an important internal resource possessed by employees themselves. This resource plays an important role in employees’ facing job demands. It interacts with work demands during times of stress, protects employees, and weakens the negative effects of stress generated by work demands. Through empirical testing, this study attempts to answer the question “What kind of employees perform more stably when faced with the pressure caused by SMUNW?” and found that employees with higher resilience are less susceptible to the impact of SMUNW, and they experience less burnout and turnover intentions. As such, this study found a new boundary condition to the influence of SMUNW and provided important implications to those who are interested in understanding the influence of SMUNW. Meanwhile, this study integrates the important concept of resilience from positive psychology to the study of social media use in today’s organizations, extending the theoretical foundation upon which technostress literature is based.

### 5.2 Practical implications

First, our findings indicate that SMUNW can lead to employee burnout and higher turnover intentions. Therefore, managers should manage employees’ use of social media. Although employees are required to utilize social media to improve work efficiency, employers should also consider the adverse effects of employees continuing to use social media to handle work after hours. Reducing social media use after work can protect employees from burning out and eventually help organizations by reducing turnover intention among their employees.

Second, our results show that burnout plays a mediating role in the effect of SMUNW on turnover intention, suggesting that paying special attention to employees’ burnout levels plays an important role. Work burnout should be investigated regularly, and employee responses to the demands created by SMUNW should be closely monitored. Furthermore, if SMUNW cannot be totally prevented due to technology developments in today’s organizations, we can utilize our knowledge of other known antecedents of burnout to partially mitigate the impairments associated with SMUNW. In other words, if SMUNW is prevalent and the results caused by this condition are unavoidable, then we should look for other paths and methods to reduce employee burnout and achieve the purpose of reducing employee turnover intentions by reducing employee burnout levels.

Third, resilience can mitigate the impact of SMUNW on burnout, thereby weakening its impact on turnover intention. This points to the importance of resilience management in the context of social media use within organizations. In the recruitment stage, it is necessary to examine the resilience of employees. It might be a good idea to employ those individuals with higher levels of resilience. Research has shown that resilient employees are better equipped to handle stress and are less likely to experience burnout (Mansfield et al., 2012). Meanwhile, targeted courses on improving resilience levels should be provided to employees in their training and development processes within organizations. Effective resilience training programs include cognitive–behavioral techniques, stress management strategies, and mindfulness practices (Robertson et al., 2015; Joyce et al., 2018). Such training programs have been demonstrated to enhance employee resilience and reduce burnout (Vanhove et al., 2016). By better equipping them to deal with technological demands during non-work hours, organizations can improve overall employee wellbeing and reduce turnover intentions.

### 5.3 Limitations and future research directions

First, cross-sectional data are used in this study so that we cannot infer causal relationships among our examined variables. Future research can consider using experimental designs to test the conclusions drawn from this study. Second, this study focuses on teachers from Shandong Province of China. Therefore, the research findings may not be generalizable to other industries and backgrounds. Future research can be conducted in different contexts to validate the robustness of the conclusions of this study. Third, in terms of moderating variables, based on the JD-R theory, this study tests the buffering effect of resilience. However, resilience is not the only type of resource an employee possesses. Future research should investigate other types of resources, such as other types of psychological resources, leadership support, and organizational policies and practices, to further investigate their buffering effects and improve our understanding of the boundary conditions of SMUNW.

## 6 Conclusion

Taken together, we found that SMUNW can induce turnover intention through increasing burnout. Moreover, resilience, as an important type of personal resource, can buffer this relationship. As the use of social media becomes increasingly common, managers should pay special attention to the detrimental effects that SMUNW can have on employees. In addition, considering the positive role of resilience in buffering the adverse effects of SMUNW, managers should pay due attention to the role of resilience in the context of technostress created by social media use.

## Data availability statement

The raw data supporting the conclusions of this article will be made available by the authors, without undue reservation.

## Ethics statement

The studies involving humans were approved by Ethics Committee, School of Public Administration and Policy, Renmin University of China. The studies were conducted in accordance with the local legislation and institutional requirements. The participants provided their written informed consent to participate in this study.

## Author contributions

ZF: Writing – review & editing, Investigation, Conceptualization. YB: Writing – review & editing, Writing – original draft, Methodology, Investigation, Conceptualization. MH: Writing – review & editing, Writing – original draft, Software, Investigation, Data curation, Conceptualization.
